# Protocol for efficient fluorescence 3′ end-labeling of native noncoding RNA domains

**DOI:** 10.1016/j.mex.2020.101148

**Published:** 2020-11-17

**Authors:** Dahlia A. Awwad, A. Rachid Rahmouni, Fareed Aboul-ela

**Affiliations:** a Center of X-Ray Determination of Structure of Matter (CXDS), Helmy Institute of Medical Sciences, Zewail City of Science and Technology, Giza, Egypt; b Centre de Biophysique Moléculaire, UPR4301 du CNRS, Orléans, France

**Keywords:** RNA, *in vitro* transcription, 3′end-labeling, Fluorescence, Native purification, Chemical tagging, Cyanine hydrazides

## Abstract

Noncoding RNAs (ncRNAs) comprise a class of versatile transcripts that are highly involved in the regulation of a wide range of biological processes. Functional long ncRNAs (> 200 nts in length) often adopt secondary structures that arise co-transcriptionally. To maintain the secondary structure elements as well as preparation homogeneity of such transcripts, native-like conditions should be maintained throughout the *in vitro* synthesis, purification and chemical tagging processes. In this optimized protocol, we describe a simple method for obtaining homogenous samples followed by chemically tagging the 3′ termini of natively-purified structured ncRNA domains that are longer than 200 nts. This protocol replaces traditional hazardous radioactive labeling with fluorescence tagging, and eliminates laborious and time consuming RNA purification and concentration steps and replaces them with straightforward recovery of RNA through centrifugal filtration, preserving the homogeneity and mono-dispersion of the preparations. The protocol provides:

• An integrative, simple and straightforward approach for synthesis, purification and labeling of structured ncRNAs whilst maintaining their secondary structure intact.
• Replacing hazardous, laborious and time-consuming radioactive labeling of RNA with much simpler fluorescence tagging, thereby facilitating potential downstream applications such as electrophoretic mobility shift assay (EMSA).
• A versatile protocol that could be applicable to a wide-range of chemical tags and in principle could be used to label DNA or RNA.

## Graphical abstract

**Figure:**
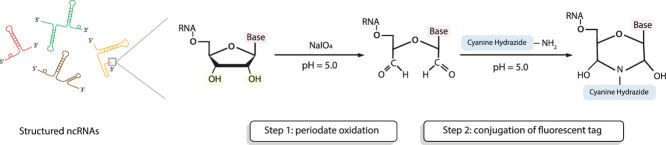

Specifications Table

**Table:** 

Subject Area	Biochemistry, Genetics and Molecular Biology
More specific subject area	RNA structural studies
Protocol name	Fluorescence 3′ end-labeling of natively purified structured ncRNA domains.
Reagents/tools	
Experimental design	1) *In vitro synthesis and native purification of ncRNA domains/fragments with lengths ranging between ~ 250 – 700 nts.* 2) *These fragments were then successfully chemically-tagged with Cy5 monohydrazide through periodate oxidation while preserving the homogeneity and native-like state of our preparations.*
Trial registration	*If applicable, include clinical trial registry and number*
Ethics	*If applicable, include ethical details e.g. Patient informed consent, Ethics Review Board-competent authority approval, animal experimentation guidelines followed etc.*
Value of the Protocol	*Describe the importance of the protocol in up to 3 bullet points:* • *An integrative, simple and straightforward approach for synthesis, purification and labeling of structured ncRNAs whilst maintaining their secondary structure intact.* • *Replacing hazardous, laborious and time-consuming radioactive labeling of RNA with much simpler fluorescence tagging, thereby facilitating potential downstream applications such as electrophoretic mobility shift assay (EMSA).* • *A versatile protocol that could be applicable to a wide-range of chemical tags and in principle could be used to label DNA or RNA.*

## Background

Non-coding RNAs (ncRNAs) have been established as important regulators in a multitude of biological processes. Functional ncRNAs, including long noncoding RNAs (lncRNAs; > 200 nts) usually adopt elaborate secondary structures that often arise co-transcriptionally. Such structures enable their functions through interactions with other macromolecules [1,2]. While preparation of RNA samples with traditional protocols that include harsh precipitation and/or freeze drying has not hindered success in many structural studies, as interest extends to longer RNAs, susceptibility to disruption of folding and/or chemical stability is becoming a greater concern. Structural and biophysical studies of longer ncRNAs require them to be synthesized and purified as homogenous species under native-like conditions, which is often a challenge to achieve *in vitro*. To overcome this, a number of protocols had been developed for native purification of *in vitro* synthesized RNA, where disruptive or denaturing steps are entirely avoided [3,4].

Labeling of RNAs with either chemical or radioactive tags is an integral part of a number of lab procedures. While radioactive tags offer the advantage of sensitivity, they are hazardous, require specialized equipment as well as specific safety precautions, and offer limited lifespan. Chemical tags, including fluorophores, provide an alternative to radioactive isotopes that is safer and easier to handle while being sufficiently sensitive for an array of downstream applications, in addition to expanding the range of these applications. For example, fluorescence resonance energy transfer (FRET) and fluorescence polarization (FP) assays rely on chemical properties of fluorescence dyes and cannot be performed with radio-labels. End-labeling of RNAs is usually preferred to in-body labeling, as it is cheaper, simpler and more versatile [5]. The use of fluorescent RNA oligonucleotides in quantitative assays has been described [6]. Periodate oxidation is a common strategy for 3′-end labeling [7,8], and is often utilized in labeling RNA oligonucleotides [9].

Here, we describe an optimized straightforward protocol for 3′ end-labeling of purified structured ncRNAs while maintaining the homogeneity of the preparations and, therefore, their potential co-transcriptional folds. The native-like conditions are maintained throughout the protocol by replacing all harsh denaturing steps of RNA recovery, such as electro-elution and salt-ethanol precipitation, with centrifugal filtration throughout the purification and labeling stages. Following this protocol, we managed to synthesize and purify ncRNA fragments with lengths ranging between ~ 250 – 700 nts. These fragments were then successfully chemically-tagged through periodate oxidation while preserving the homogeneity and native-like state of our preparations.

## Experimental design

As a general rule, working with RNA requires maintaining RNase-free conditions at all times. Therefore, all solutions should be prepared with Diethyl Pyrocarbonate (DEPC) - treated water, and all consumables and glassware should be sterilized with DEPC-water and autoclaved. The most widely-accepted recipe for preparing DEPC- treated water includes addition of 0.1% DEPC, incubation for 1 hour at 37 °C followed by autoclaving for several hours/overnight to ensure the elimination of ethanol and other by-products of DEPC treatment that might interfere with enzymatic reactions.

### Enzymatic synthesis of RNA by *in vitro* transcription

Generally, templates of RNAs of interest should be inserted into a suitable vector so that they are immediately flanked at the 5′ end by a promoter sequence (*e.g.* T7 or SP6 promoter) and at the 3′ end by a unique restriction site that enables linearizing the plasmid prior to *in vitro* transcription [10]. Alternatively, template sequences could be produced using PCR so that the T7 promoter sequence is added immediately at the 5` end, as previously described [11].

**Fig. 1:**
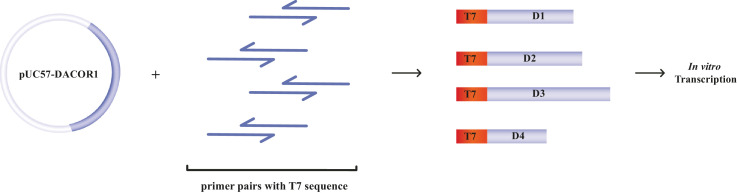
Preparation of DNA templates for *in vitro* transcription: To prepare the ncRNA domains (blue) we utilized throughout this protocol, we used the long noncoding RNA DACOR1 as a template for PCR to generate four smaller fragments (designated D1, D2, D3 and D4) using four pairs of unique primers. The flanking sequence of the T7 promoter (red) is inserted at the 5`- end of each template for all of the four fragments.

The ncRNAs utilized in this protocol are four overlapping fragments covering the whole length of the long noncoding RNA DACOR1 [12]. For *in vitro* transcription, template sequences of these fragments were generated using PCR, with the T7 promoter sequence introduced immediately upstream of each fragment. The sequence of DACOR1 was generated synthetically (GenScript) and carried over as an insert in the pUC57 vector between the unique restriction sites of *Bam*HI and *Sma1* (Fig. 1).

Transcripts of isolated fragments were generated with *in vitro* transcription using T7 polymerase that was produced in-house and kindly provided by Marc Boudvillain (CBM, CNRS, Orleans). Sequences that were produced by PCR and purified with a PCR cleanup kit (Qiagen) were used as templates. *In vitro* transcription was carried out with 6 µg/mL of template DNA and 10x transcription buffer (15 mM MgCl_2_, 0.8 M Tris-HCl pH 8.1, 40 mM spermidine, 0.2% Triton X-100, 100 mM DTT) in addition to T7 polymerase, 2 mM of each of the four rNTPs (Invitrogen) and RiboLock™ RNase Inhibitor (40 U/µL; Thermo Scientific) followed by incubation for 3 hours at 37 °C. Scavenging the pyrophosphates that are generated as a byproduct of the T7 polymerization activity might increase the transcription yield. This can be achieved by adding inorganic pyrophosphatase (iPPases) to the transcription reactions [11].

We found that incubating the transcription reactions for longer than 4 hours might result in producing abortive transcripts. It is worth-noting as well that the concentration of magnesium ions in the transcription mixtures could substantially impact the T7 activity as well as the homogeneity of the RNA preparation, and therefore should be optimized for every ncRNA fragment [13,14]. Importantly, excessive Mg^+2^ concentrations could lead to the formation of non-specific aggregates.

### Native purification of synthesized RNA

RNA purification and recovery was performed under native-like conditions maintaining the samples at room temperature at all time, as previously described [4,13]. Throughout the protocol, recovery and purification of RNA are performed using centrifugal filtration (*see following sections*), such as the commercially-available Amicon^Ⓡ^ Ultra Centrifugal Filters (Millipore). The Amicon^Ⓡ^ filters are commonly utilized for the concentration and desalting of protein samples prior to various chromatography applications, such as HPLC, and they are ideal for removal of solutes, nucleotides, primers and other low molecular weight components from reactions. This step allows for buffer exchange as well as filtering out unreacted NTPs, digested proteins and DNA fragments as well as short abortive transcripts from our RNA preparations.

Following transcription, the reactions were supplemented with 1.2 mM CaCl_2_ and DNase I (2000 U/ml; New England BioLabs) and incubated for 30 min at 37 °C, and subsequently with proteinase K (20 mg/ml; Thermo Scientific) and incubated for 45 min at 37 °C. Immediately afterwards, reactions were diluted with a HEPES-based filtration buffer (HEPES-FB; 100 mM NH_4_Ac, 8 mM HEPES pH=7.3, 0.1 mM Na-EDTA pH=8.5) and applied to Amicon Ultra-4 centrifugal filters (Millipore; 30 kDa molecular weight cut-off). Centrifugation was done at 4000 *g* for 15 min at room temperature. Following filtration, Nanodrop measurements were taken to ensure sample purity and homogeneity as well as sufficient elimination of enzymes and other proteins as represented by an A_260_/A_280_ nm absorbance ratio that ranges between 2.0 and 2.5.

It is important to maintain native conditions throughout the whole protocol. Thus, RNA purification and recovery steps that involve salt-ethanol precipitation, freezing or extreme agitation of RNA should be avoided entirely in order to keep the secondary structure of the RNA domains intact. In addition, digestion of proteins with proteinase K followed by centrifugal filtration is often sufficient for the removal of enzymes such as T7 polymerase [4], eliminating the need for phenol-chloroform RNA purification.

**Fig. 2:**
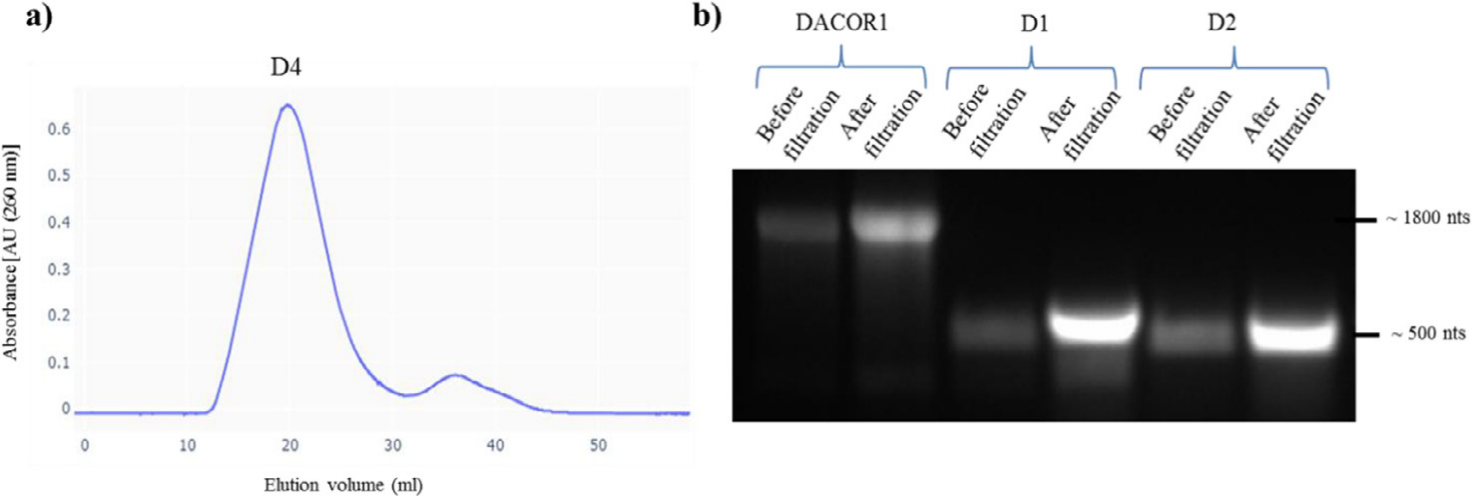
Examining the homogeneity of RNA preparations. a) Example SEC chromatogram of the fragment D4, represented as UV absorbance (AU) *versus* elution volume (ml) and showing single sharp peak, which indicates homogeneity and mono-dispersion of the RNA preparation. b) Transcription reactions of whole-length DACOR1, D1 and D2 (before and after Amicon filtration) that were resolved on 1% native agarose gel and visualized by UV following staining with ethidium bromide.

The integrity and homogeneity of the reactions should be consistently monitored throughout this stage through gel electrophoresis using either native polyacrylamide or native agarose gel and visualization by ethidium bromide staining. A well-defined single band for each RNA species could serve as a preliminary indicator to the homogeneity of the preparation (Fig. 2b). Additionally, it is recommended at this stage to examine the homogeneity of the RNA preparations using size exclusion chromatography (SEC). In this protocol, as a quality control step following purification on centrifugal filters, SEC was performed at room temperature using HEPES-FB as a running buffer. For each transcript, Tricorn columns (GEHealthcare) were packed with Sephacryl S300 medium. Before each run, we washed the columns with 4 column volumes (CVs) of DEPC-treated water and equilibrated them with 4 CVs of HEPES-FB solution (Fig. 2a).

Nonetheless, we found that using a processive T7 combined with multiple steps of centrifugal filtration was often enough to obtain concentrated homogenous samples, eliminating the need for further purification. It is also worth noting that homogenous preparations of the whole-length DACOR1 lncRNA molecule (~1800 nts) were obtained following the same synthesis and purification protocol (Fig. 2b).

### Fluorescence 3′- end labeling of RNA

**Fig. 3:**
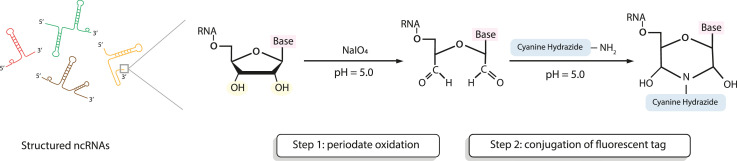
Labeling of structured ncRNAs at the 3′ terminus through periodate oxidation. Step 1: the vicinal hydroxyl groups of the 3′ terminal ribose is subjected to periodate oxidation converting it into a dialdehyde. Step 2: The added hydrazide salt reacts with the aldehyde group leading to conjugation of the chemical tag to the RNA 3′ terminus.

The strategy of 3′ end-labeling of RNA through periodate oxidation has long been established [5,8]. It mainly comprises two major steps; the first step is oxidizing the 3′ terminal ribose sugar using sodium periodate into a reactive aldehyde group. The second step is conjugating an aldehyde-reactive chemical dye, such as cyanine monohydrazide or fluorescein 5-thiosemicarbazide (FTSC), to the oxidized 3′ terminus of the RNA (Fig. 3).

Nonetheless, typical protocols of 3′ end-labeling do not generally consider RNA secondary structure and might involve common handling or purification steps that are disruptive to the secondary structure elements of the RNA samples, rendering such samples potentially unsuitable for secondary structural analysis. The fluorescence end-labeling protocol described in this section maintains the RNA secondary structure intact. This optimized protocol eliminates disruptive steps (for example, concentrating RNA through salt-ethanol precipitation) and instead replaces them with direct filtration using centrifugal filters. Fluorescence dyes that could be used in this method are generally cyanine hydrazides, namely cyanine 3 (Cy3) and cyanine 5 (Cy5). For our RNA domains, we utilized Cy5 throughout this protocol.

#### Periodate oxidation of RNA 3′ terminus

Unlike other labeling methods, such as radioactive tags, periodate oxidation is specific for RNA because it requires vicinal hydroxyl groups. Additionally, a wide range of aldehyde-reactive fluorophores could be used. Immediately after RNA recovery, oxidation reactions were assembled as described in Table 1. The reactions were incubated at room temperature for 90 minutes in the dark. Typically, reactions are done in 100 µl final volume. However, the final volumes of reactions could be up- or downscaled as needed with adjusting the volumes of the components to maintain the final concentration.

**Table 1:** components of the RNA 3′ oxidation reactions.

Component	Final Concentration
RNA	> 7.5 µM
Sodium Acetate (pH= 5.0)	100 mM
Sodium periodate (NaIO_4_) *Freshly prepared*	100 mM
Add DEPC—H_2_O up to a final reaction volume of 100 µl.	

After incubation, we proceeded immediately to Amicon filtration to eliminate the NaIO_4_ of the reaction. We found that the Amicon filtration was sufficient to stop the oxidation reaction, rendering the traditional step of precipitating the IO^4−^ ions out of the solution by adding another monovalent salt unnecessary. For optimal concentration of our RNA preparations, we used the Amicon Ultra-0.5 centrifugal filters (Millipore; 50 kDa molecular weight cut-off) and centrifuged for 30 minutes at 14,000 g.

An additional advantage for using gravity-based or centrifugation- based filtration methods is the elimination of further RNA precipitation steps that might involve salt-ethanol or prolonged incubation on ice, both of which were found to disrupt the secondary structure of RNA and therefore should be avoided entirely when preparing RNA samples for secondary structural studies. It is worth-mentioning that lyophilization of RNA samples should also be avoided, as it probably leads to the formation of aggregates that might interfere with further methods [15]. In addition to preserving the secondary structure elements, eliminating ethanol precipitation diminishes the possibility that traces of ethanol in the RNA preparations would interfere with the dye conjugation reaction in the next labeling step (see section 3.2).

#### Conjugation of chemical tag (Cyanine-5 hydrazide)

To prepare Cy5 labeling solution from the Cy5 hydrazide salt stock, a vast excess of the dye salt is dissolved in neat DMSO to prepare a solution of 5 mM final concentration. The dye solution should then be divided into aliquots of 10 µl each and dried by centrifugation under vacuum (Speed-Vac). Aliquots are stored at −20 °C in the dark and, before assembling a conjugation reaction, an aliquot should be re-suspended in 10 µl of neat DMSO.

Immediately after recovering the oxidized RNA, assemble the conjugation reactions as described in Table 2. Incubate the reactions at room temperature for 4 hours or overnight in the dark under gentle agitation. Immediately after incubation is over, excess dye is eliminated and labeled RNA is recovered using Amicon Ultra-0.5 centrifugal filters (Millipore; 50 kDa molecular weight cut-off) by centrifuging for 20 minutes at 14,000 g.

**Table 2:** components of the labeling reaction.

Component	Final Concentration
Oxidized RNA	> 12 µM
Sodium Acetate (pH=5.0)	100 mM
Cy5 hydrazide solution	1.7 mM
Add DEPC—H_2_O up to a final reaction volume of 30 µl.	

**Fig. 4:**
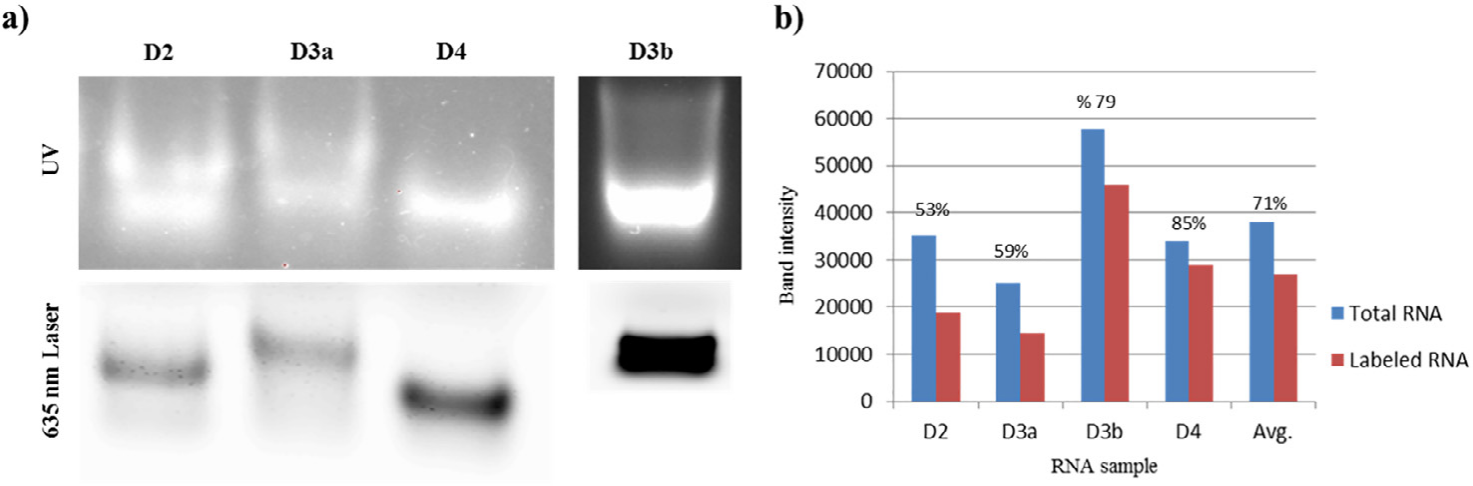
RNA preparations labeled with Cy-5. **a)** Native RNA domains (D2, D3 and D4) end-labeled with Cy5 and resolved on 1% agarose gel from two different trials. The total RNA as visualized by ethidium bromide staining using UV is shown in the upper UV panel. The lower panel shows the Cy-5 labeled RNA fraction of each sample visualized with 635 nm laser. **b)** The ratios of labeled RNA/ total RNA calculated from image analysis of relative band intensities and used as an estimate to the average labeling efficiency. Samples D2 and D3a were stored at room temperature for approximately 72 hours before labeling, whereas samples D3b and D4 were stored for less than 24 hours.

Similar to the step of recovering the oxidized RNA, purifying and concentrating the labeled RNA by means of Amicon centrifugal filters here replaces the traditional precipitation of RNA using salt-ethanol precipitation/ freezing methods. The integrity and purity of labeled RNA is examined by resolving the samples on either native polyacrylamide or native agarose gel, and visualized using an appropriate imaging system/ machine. We examined our labeled RNA samples (D2, D3 and D4) by resolving on 1% native agarose gel for ~ 1 hour. The wet gels were then visualized using a Typhoon FLA-9500 machine equipped with a 635 nm excitation laser source giving rise to an emission peak of the Cy-5 at wavelength of around 670 nm (LPR filter). Images were analyzed by the ImageQuant and Fiji Imagej software. For the total RNA, gels were stained with ethidium bromide and visualized using a UV-based gel documentation system (Bio-Rad). Image analysis of band intensities to estimate labeling efficiency as average ratios of (labeled RNA/total RNA) returned an estimated percentage ranging between 70 and 75% (Fig. 4). Although samples were stored at room temperature to preserve folding, RNA preparations that were stored at room temperature for longer than 72 hours displayed an apparent decrease in efficacy of the labeling to around 55–60% (Fig. 4; D2 and D3a).

## Conclusion and value of the protocol

In this optimized protocol, we describe a simple method for chemically tagging the 3′ end of natively-purified structured ncRNA domains without compromising their secondary structures. Moreover, we eliminate laborious and time consuming RNA purification and concentration steps and replace them with straightforward recovery of RNA through Amicon centrifugal filtration. The whole protocol can be done on the bench top and does not require specialized equipment or specific safety precautions, compared to radioactive labeling. The resulted chemically-tagged ncRNAs could be used in a multitude of downstream applications; for example, to detect the binding potential to proteins using electrophoretic mobility shift assay (EMSA), and could be particularly useful in quantitative assays that depend on the properties of fluorescence dyes such as FRET and FP assays. Wet-gel scanning and detection could be performed relatively easily, using equipment that is readily available such as Typhoon and Fuji Fluorescent Image Analyzer (FLA-5000 or FLA-9000).

Overall, replacing traditional hazardous radioactive labeling with fluorescence labeling facilitates the handling process of RNA and eliminates the need for specialized equipment as well as elaborate safety precautions. The activity of the recovered labeled ncRNA domains could subsequently be closely examined in their native conformation with a versatile wide-range of downstream experiments such as EMSA. This protocol is applicable to several types of chemical tags and, although we mainly use ncRNA fragments throughout the protocol, could be used for modifying both DNA and RNA.

## Declaration of Competing Interest

The authors declare that they have no known competing financial interests or personal relationships that could have appeared to influence the work reported in this paper.

## References

[1] Lai D., Proctor J.R., Meyer I.M (2013). On the importance of cotranscriptional RNA structure formation. RNA.

[2] Chen Q., Chen Y.P.P. (2011). Modeling conserved structure patterns for functional noncoding RNA. IEEE Trans. Biomed. Eng..

[3] Edelmann F.T., Niedner A., Niessing D (2014). Production of pure and functional RNA for *in vitro* reconstitution experiments. Methods.

[4] Chillón I., Marcia M., Legiewicz M., Liu F., Somarowthu S., Pyle A.M. Native purification and analysis of long RNAs. In: Methods in Enzymology. 2015. p. 3–37. 10.1016/bs.mie.2015.01.008 PMC4477701 26068736

[5] Zearfoss N.R., Ryder S.P. (2012). End-labeling oligonucleotides with chemical tags after synthesis. Methods Mol. Biol..

[6] Pagano J.M., Clingman C.C., Ryder S.P (2011). Quantitative approaches to monitor protein-nucleic acid interactions using fluorescent probes. RNA.

[7] Qin P.Z., Pyle A.M. (1999). Site-specific labeling of RNA with fluorophores and other structural probes. Methods A Companion Methods Enzymol.

[8] Reines S.A., Cantor C.R. (1974). New fluorescent hydrazide reagents for the oxidized 3’-terminus of RNA. Nucl. Acids Res..

[9] Qiu C., Liu W.Y., Xu Y.Z (2015). Fluorescence labeling of short RNA by oxidation at the 3′-End. Methods Mol. Biol..

[10] Milligan J.F., Groebe D.R., Witherell G.W., Uhlenbeck O.C (1987). Oligoribonucleotide synthesis using T7 RNA polymerase and synthetic DNA templates. Nucl. Acids Res..

[11] Beckert B., Masquida B. (2011). Synthesis of RNA by *in vitro* transcription. Methods Mol. Biol..

[12] Merry C.R., Forrest M.E., Sabers J.N., Beard L., Gao X.H., Hatzoglou M. (2015). DNMT1-associated long non-coding RNAs regulate global gene expression and DNA methylation in colon cancer. Hum. Mol. Genet..

[13] Somarowthu S., Legiewicz M., Chillón I., Marcia M., Liu F., Pyle A.M (2015). HOTAIR forms an intricate and modular secondary structure. Mol. Cell.

[14] Brunelle J.L., Green R.*In vitro* transcription from plasmid or PCR-amplified DNA. In: Methods in Enzymology. 2013. p. 101–14. 10.1016/B978-0-12-420037-1.00005-1 24034317

[15] Lentzen G., Klinck R., Matassova N., Aboul-Ela F., Murchie A.I.H (2003). Structural basis for contrasting activities of ribosome binding thiazole antibiotics. Chem. Biol..

